# Hepatitis B Virus infection in HIV-positive population in Brazil: results of a survey in the state of Mato Grosso and a comparative analysis with other regions of Brazil

**DOI:** 10.1186/1471-2334-6-34

**Published:** 2006-02-25

**Authors:** Rui Alberto Roldão de Almeida Pereira, Aparecida Duarte Hg Mussi, Vergínia Correa de Azevedo e Silva, Francisco José Dutra Souto

**Affiliations:** 1 Faculdade de Medicina da Universidade de Cuiabá, Cuiabá, MT, Brazil; 2 Laboratório Central de Mato Grosso, Cuiabá, MT, Brazil; 3 Faculdade de Ciências Médicas, Universidade Federal de Mato Grosso, Cuiabá, MT, Brazil

## Abstract

**Background:**

End-stage liver disease is currently a major concern among HIV-positive individuals due to co-infection with hepatotropic virus. Hepatitis C has been pointed out as a remarkable factor for that. More recently, hepatitis B virus (HBV) infection has also been found to play a role on liver disease in this population. HIV-HBV co-infection prevalence remains largely unknown in vast areas of Brazil. The objective of the present study was to estimate the prevalence of HBV and HDV infection in HIV-infected subjects living in the state of Mato Grosso, in the Central region of Brazil, and compare it to other Brazilian studies. We also assess epidemiologic data regarding risk factors and vaccinal status.

**Methods:**

HIV-positive individuals followed at the Central Laboratory of the Department of Public Health of Mato Grosso in the city of Cuiabá composed the sample. Participants answered a specific questionnaire and had a blood sample taken and tested for serologic markers.

**Results:**

A thousand individuals were interviewed and tested for HBsAg, anti-HBc, anti-HBs and anti-HDV if positive for HBsAg. Measurements of CD4 and viral load for HIV-1 were also performed. Overall prevalence of HBV exposure (anti-HBc +ve) was 40.0%, and 3.7% for HBsAg. This prevalence data was similar or slightly lower than for other Brazilian regions, which ranged from 40% and 3% to 71% and 24%, respectively. Testing for anti-HDV in the 37 HBsAg positive patients was positive in only one subject. Factors that showed independent association with HBV exposure, after adjustment, were: male gender, older age groups, tattooing, and reporting more than ten sexual partners throughout life (p < 0.01). Eighty-one (27.5%) out of 291 HBV-unexposed individuals who reported vaccination were anti-HBs positive. Anti-HBs prevalence was higher among those who had higher levels of CD4 by multivariate analysis.

**Conclusion:**

Our data showed HBV infection prevalence similar or slightly lower than that reported in other regions of Brazil. In addition, our data revealed a less important role for drug injection in the spread of HIV and HBV in Mato Grosso compared to other regions of the country. The high rate of non-vaccinated subjects among this HBV-unexposed, HIV-infected population is a matter of considerable health concern in this region. The relationship between CD4 levels and HBV vaccine response found in the present study reinforces the need of keeping health care workers alert to this issue.

## Background

Hepatitis B Virus (HBV) infection and its associated complications (cirrhosis and hepatocellular carcinoma) are the leading cause of chronic liver disease worldwide. An estimated one third of the global population has been infected with the virus and more than 350 million people are chronic carriers [1].

Prevalence of HBV infection is heterogeneous, reaching a high carrier rate in certain countries and among some subpopulations. Brazil is a country with intermediate prevalence, having high and low prevalence in distinct geographical areas [2].

Among HIV-infected individuals, HBV infection prevalence is approximately ten times higher than in the general population, due to shared routes of transmission [3]. Although uncommon until recently, due to the low life expectancy of HIV-infected persons, clinical manifestations of chronic hepatitis B in the HAART era are becoming more frequent following the increased survival of these patients [4].

Chronic HBV infection appears to have its natural progression modified by HIV coinfection, with significantly increased mortality attributable to liver disease in co-infected patients, compared with those with HBV alone [5]. Decreased clearance rates of HBsAg and HBeAg, reactivation of HBV, higher levels of HBV-DNA, and lower ALT levels are some of the most important findings that differentiate HIV-HBV co-infected individuals from those infected only with HBV [6-8]. Consequently, it is of pivotal importance to determine the prevalence of HIV-HBV co-infection in order to estimate chronic liver disease burden due to HBV among HIV-infected patients.

In Brazil, data on HBV infection among HIV-positive subjects is still scarce. However, some studies, conducted mainly in the South and Southeast regions (the most developed and populous parts of the country), have reported prevalence of HIV-HBV co-infection ranging from 5.3 to 24.3% [9-13].

There is a lack of information on this issue in Central Brazil, a vast region located in the innermost part of South America. Furthermore, data on hepatitis D virus infection in HIV-HBV co-infected patients of this region is also not available.

The goal of the present study was to estimate the prevalence of HBV and HDV infection in HIV-infected patients living in the state of Mato Grosso – Central Brazil – treated at the STD/AIDS Reference Unit in the city of Cuiabá, comparing it with available data from other Brazilian regions. Epidemiological data regarding risk factors and vaccinal status was also assessed.

## Methods

A cross-sectional study was designed with the goal of assessing epidemiological and serologic data. In the state of Mato Grosso, virologic and immunologic assessment and medical assistance of HIV-infected subjects are provided by the Department of Public Health at the Central Laboratory and STD/AIDS Reference Unit, respectively. Consequently, almost all known HIV-infected persons of Mato Grosso are referred to this unit to perform viral load and CD4 measurements, including most of the subjects treated at private clinics, at which time they were invited to participate in the study. The survey was conducted over a six-month period, in view of the recommendation that these exams be performed every 4 to 6 months.

Individuals were considered eligible if they were over 17 years of age and agreed to answer a specific questionnaire and have a blood sample taken and tested for serologic markers.

A standard questionnaire was applied to obtain information on demographics, risk factors for HIV and HBV infection, previous sexually transmitted diseases, and vaccinal status for HBV.

Blood samples were tested by enzyme immunoassay for HBV surface antigen, HBsAg (EIE-HBsAg^® ^– Bio-Manguinhos, Rio de Janeiro, Brazil), for antibodies against core antigen (anti-HBc), against HBsAg (anti-HBs) and against HDV (anti-HDV) – (Hepanostika^® ^Microelisa system anti-HBc Uni-Form, anti-HBs, and HDV respectively – Biomerieux, Boxtel, The Netherlands).

T-lymphocyte subsets were determined by flow cytometry technique (Beckton-Dickinson, NJ, USA). HIV1-RNA detection and measurement were performed by NASBA (Biomèrieux, Boxtel, The Netherlands).

The Ethics Research Board at the Mato Grosso Federal University approved the protocol used in the present study. All surveyed individuals who agreed to participate were asked to sign informed consent form. Serologic marker results were used to assistant physicians in order to provide adequate measures when indicated.

The statistical analysis was based on overall chi-square test with Yates' correction and Fisher's exact test. When appropriate, crude odds ratio and the confidence interval were calculated to determine the strength of association. Multiple logistic regression models were constructed by SPSS 12.0 software to assess the independent effect of variables.

In order to compare our results with available data from other Brazilian regions, we performed a search in the Medline (National Library of Medicine, US) and LILACS (Latin America and Caribbean Health Sciences Information System, PAHO) electronic databases. Our search strategy was to combine the terms "brazil," "hiv," "hepatitis," and "prevalence" in several ways.

## Results

Between January 2004 and June 2004, 1,025 individuals were approached. Eight refused to participate and 17 were excluded from analysis due to missing data at the interview or technical difficulties in performing a blood test (insufficient or inappropriate blood sample). A thousand individuals were interviewed and tested, corresponding to approximately 80% of the population in regular follow-up with CD4/CD8 and viral load measurements at Central Laboratory. Patients originated mainly from Cuiabá and surrounding counties (71.5%) and the remainder (28.5%) lived in more remote parts of the state.

Five hundred thirty were male. The mean age of the patients studied was 37.2 years (SD 10.1 and median 36.0). On average, women were younger than men (34.6 ± 9.7 vs. 39.4 ± 10.0 yrs). Table 1 shows the demographic characteristics of the patients.

**Table 1:** Demographic and epidemiological characteristics of HIV-positive individuals in the state of Mato Grosso, Central Brazil.

Characteristic	N (%)	Male N (%)	Female N (%)	P
Total	1000 (100.0)	530 (53.0)	470 (47.0)	
				
Age group (in yrs.)				
17–30	268 (26.8)	100 (18.8)	168 (35.7)	0.000
31–40	408 (40.8)	213 (40.2)	195 (41.5)	0.5
41–50	230 (23.0)	151 (28.5)	79 (16.8)	0.000
51–77	94 (9.4)	66 (12.4)	28 (5.9)	0.6
				
Supposed route of HIV infection				
Unprotected sex	577 (58.8)	403 (76.0)	174 (37.0)	0.000
HIV+ve partner	322 (32.8)	66 (13.2)	256 (54.4)	0.000
Blood transfusion	56 (5.7)	30 (5.6)	26 (5.5)	NS
IV-drugs	24 (2.4)	21 (4.0)	3 (0.6)	0.001
				
Number of sexual partners				
1–5	379 (38.1)	98 (18.4)	281 (59.7)	
6–10	164 (16.5)	86 (16.2)	78 (16.6)	
> 10	451(45.3)	341 (64.3)	110 (23.4)	0.000*
				
STD				
Yes	339 (34.2)	266 (50.7)	73 (15.6)	0.000
No	653 (65.8)	259 (49.3)	394 (84.4)	
				
Tattooing				
Yes	134 (13.5)	86 (16.4)	48 (10.3)	<0.01
No	857 (86.5)	438 (83.6)	419 (89.7)	
				
Vaccination against hepatitis				
Yes	464 (47.6)	239 (46.7)	225 (48.6)	0.6
No	511 (52.4)	273 (53.3)	238 (51.4)	
				
Antiretroviral treatment				
Yes	716 (72.0)	402 (76.3)	314 (67.1)	<0.05
No	279 (28.0)	125 (23.7)	154 (32.9)	
				
CD4 level (cells/mm^3^)				
0–200	255 (25.5)	143 (27.0)	112 (23.8)	
201–350	288 (28.8)	141 (26.6)	147 (31.3)	
> 351	457 (45.7)	246 (46.4)	211 (44.9)	0.7*
				
Viral Load (copies/mm^3^)				
< 80	386 (38.6)	224 (42.2)	162 (34.5)	
81–10,000	242 (24.2)	114 (21.5)	128 (27.2)	
10,001–100,000	234 (23.4)	115 (21.7)	119 (25.3)	
> 100,000	138 (13.8)	77 (14.6)	61 (13.0)	0.2*

* Chi-square for linear trend

Unsafe sexual activity (defined as having unprotected sex with partners of unknown serologic status for HIV) was the more commonly self-reported route of HIV infection (58.8%), followed by monogamous relationship with a lately identified HIV-infected partner (32.8%), history of blood-product transfusion (5.7%), and use of illicit-IV-drugs (2.4%). Only 0.2% of the individuals denied belonging to any of the above risk groups. Tattoos were present in 13.5% of the sample. Risk factors differed according to the distribution by gender: 55.8% of the women reported an HIV-infected partner compared to 12.6% of men. Males (77.2%) reported unsafe sexual activity more frequently than females (37.9%, p < 0.0001).

Sex-related exposure, as determined by the total number of sexual partners through life, showed 38.1% having 1 to 5 partners, 16.5% having 6 to 10 partners, and 45.3% having more than 10 partners.

Sexually transmitted diseases were reported by 34.2%, with males accounting for 78.5% of the occurrences (p < 0.0001). Genital lesions were mentioned by 49.8%, and syphilis by 21.2% of the individuals reporting STD. Urethral discharge was reported by 68.1% of the men who had STD.

A total of 716 individuals (72%) were currently undergoing antiretroviral treatment and 279 (28%) reported no treatment.

There was evidence of HBV infection in 400/1000 (40%) subjects (anti-HBc +) and 37 (3.7%) were reactive for HBsAg. 263 (72.4%) out of 363 patients with previous exposure were also anti-HBs positive.

Of the remaining 600 individuals, 109 were positive for anti-HBs alone. Testing for anti-HDV in the 37 HBsAg positive patients was positive in only one subject (Table 2).

**Table 2:** HBV markers in HIV-positive individuals in the state of Mato Grosso, Central Brazil.

Marker	n (%)	CI 95%
HBV exposed (Anti-HBc +ve)	400 (40.0)	(36.9 – 43.1)
HBsAg +ve	37 (3.7)	(2.6 – 5.2)
Previous infection	363 (36.3)	(33.3 – 39.4)
Anti-HBc + Anti HBs	263 (72.4)	(67.5 – 76.9)
Anti-HBc alone	100 (27.6)	(23.1 – 32.5)
		
HBV non-exposed	600 (60.0)	(56.9 – 63.0)
Anti-HBs alone	109 (18.2)	(15.2 – 21.5)

* Chi-square for linear trend

A multivariate analysis demonstrated that HBV exposure was independently associated with the following factors: male gender, older age groups, tattooing, and reporting more than ten sexual partners throughout life (Table 3). Reactivity for HBsAg was more common among males, but this association was not confirmed after age adjustment. After logistic regression, no association remained positive for HBsAg carrier state.

**Table 3:** Risk factors associated with HBV infection after adjusted analysis.

Risk factors	OR	CI 95%	P*
Male gender	1.6	1.2–2.1	0.00
			
Age group			
17–30	1.0	-	-
31–40	2.0	1.4–2.8	0.00
41–50	2.7	1.8–4.0	0.00
51–77	3.9	2.3–6.6	0.00
			
Sexual exposure			
0 – 9 partners	1.0	-	-
> 10 partners	1.4	1.1–1.9	0.01
			
Tattooing	1.6	1.1–2.4	0.01
			
IV drugs	2.3	0.9–5.7	0.07

* P-value of chi-square

Vaccination against hepatitis was reported by 47.6% of subjects (defined as hepatitis B vaccine by 83.8% of those vaccinated). HBV vaccine was indicated by the assistant physician in 75.4% of the individuals submitted to immunization. Eighty-one (27.5%) out of 291 HBV-unexposed individuals who reported to had taken the vaccine were anti-HBs positive. 294 (50.2%) of 585 HBV susceptible subjects never took the vaccine.

A statistical analysis was also performed regarding vaccination against HBV and seroconversion rates for anti-HBs. A univariate analysis showed significantly higher rates of anti-HBs in the patients with higher levels of CD4 who reported vaccination and were without serologic evidence of prior contact with HBV (anti-HBc negative). This association remained unchanged after multivariate analysis, showing better performance of vaccinal response among individuals with > 350 cells/mm^3 ^(Table 4).

**Table 4:** Analysis of vaccinal response (Anti-HBs+) and CD4 level among HBV-unexposed individuals*.

CD4+ level	Anti-HBs +/total (%)	OR	CI 95%	p
0–200	6/46 (13.0)	1.0	-	-
201–350	16/87 (18.3)	1.5	0.5 – 4.2	0.4
> 350	59/158 (37.3)	4.2	1.6 – 10.7	0.02
Total	81/291 (100.0)			

*Adjusted for age group and gender.

We have found five articles assessing prevalence of HIV-HBV co-infection in Brazilian populations. Comparison with the present study revealed similar anti-HBc and HBsAg prevalence with data from the Southeast region. Prevalence from the North and South regions were higher than in our study (Table 5).

**Table 5:** Prevalence of HBV infection in HIV-positive populations reported in different Brazilian studies.

Region (Main city)	n	Anti-HBc +ve	HBsAg +ve	IV drugs	Year	Reference
North (Belém)	406	51.0% (46.0–56.0)	7.9% (5.5–11.1)	10.6% (7.9–14.1)	2000	13
Central (Cuiabá)	1,000	40.0% (37.0–43.1)	3.7% (2.7–5.1)	2.4% (1.6–3.6)	2004	Present study
Southeast (Campinas)	226	44.0% (37.5–50.7)	5.3% (2.9–9.3)	29.0% (23.3–35.5)	1995	11
" (Ribeirão Preto)	401	40.9% (36.1–45.9)	8.5% (6.0–11.8)	22.2% (18.3–26.6)	2002	10
" (São Paulo)	1,693	38.6% (36.3–41.0)	5.7% (4.7–6.9)	-	1996	12
South (Florianópolis)	93	71.2% (60.7–79.9)	24.3% (16.3–34.5)	36.5% (26.9–47.2)	1999	9

## Discussion

High HBV infection rates have been described among HIV-infected population worldwide [5,14,15]. The risk of HBV-associated end-stage liver disease seems to be increased in the setting of HIV-co-infection [3,5]. Consequently, it is very important to estimate its prevalence regionally.

In our study, we took advantage of the official policy of the Brazilian government for the health care for HIV-positive subjects. Such policy provides, in addition to HAART supply, measurement of HIV-RNA and CD4 cell count every four to six months. In the present study, the survey period lasted six months in order to include the vast majority of patients when they returned to the Central Laboratory for their next viral and immunological evaluation. Consequently, the 1,000 individuals assessed represent almost all the HIV-positive population, diagnosed and followed-up on in the state of Mato Grosso.

The overall prevalence rate of HBV infection in the studied population was 40.0%, higher than that found among blood donors in Mato Grosso (approximately 10%, according to Mato Grosso State public blood bank – Hemomat Official Report 1999–2002). Regarding the rest of Brazil, the present study showed similar anti-HBc prevalence when compared with data from the Southeast region. Prevalence from the North and South regions were higher than the one found in our study. Other authors have reported prevalence of HBV markers among HIV-positive individuals ranging from 36% to 50% (Southeast), 46.0% to 56% (North), and 60% to 79% (South), [9-13]. The rate of HBV carriers for the present study was also comparable to the prevalence in the Southeast and lower than in the North and South regions. However, data from the South region was based on a very small sample [9]. Furthermore the very impressive presence of IVD users in the South region suggests that a selection bias has occurred (Table 5).

A statistical association regarding risk factors for HBV infection showed higher prevalence in older age groups, a well-known and widely observed fact, due to the increasing risk of exposure with time. A stronger association with the male gender was also observed, as described previously in many populational groups, probably because of more frequent exposure to the risk factors involved in transmission, particularly sexual contact with a greater number of partners.

More than 10 sexual partners was another independent risk factor for HBV infection in our study, reinforcing the importance of this transmission route, as already observed in the general population.

Two classic risk factors for blood-borne diseases were associated to HBV markers: tattooing (p = 0.01) and the use of IV drugs, although the statistical significance was only marginal (p = 0.07) in the logistic model for the latter. This lack of significance probably occurred due to the small number of participants reporting drug injection and because both variables were frequently present in the same subjects (50% of tattooed subjects were also IV drug users). There is a striking difference between the rate of reported intravenous drug use in the present study (2.7%) and that of other studies conducted in the South and Southeast regions of Brazil (22.1%, 29.0%, 36.5%) [9-12]. The use of IV drugs was also associated to a higher prevalence of HBV infection in these studies, reflecting the higher transmissibility of HBV through direct blood contact while sharing syringes and needles. In spite of the possibility of some participants having concealed information about IV drug use, the difference between the prevalence in Mato Grosso and other Brazilian regions was quite impressive and it seems to influence the prevalence of HBV markers. We believe that the HIV-positive population of Mato Grosso has a different epidemiological profile. In fact, low-income adult women in monogamous relationships composed the largest portion of the sample. This is not the usual profile of IV drug users.

The sexual route seems to be much more implicated in HIV spread in Mato Grosso than the parenteral route. This finding may play a role in the lower prevalence of HBV markers among the HIV-positive population of Mato Grosso.

Only one (2.7%) HBsAg carrier had anti-HDV positivity. It is considered a low HDV infection prevalence and differs from data of Brazilian surveys conducted in the general population in the Amazonian region [16-18]. A high HDV infection prevalence was recently shown in the portion of the state of Mato Grosso that belongs to the Amazonian region [18]. Nevertheless, this alarming figure was not found among our HIV-HBV co-infected sample, since HIV has a more urban pattern in Brazil.

Vaccination is a widely accepted strategy for lowering the incidence of HBV infection in HIV-infected individuals [19]. Given the increased rate of chronic hepatic disease and more unfavorable outcome in this subpopulation, physicians must be aware of the importance of detecting carriers and immunizing susceptible individuals.

In our study, 47.6% of those interviewed reported vaccination against hepatitis. Most (83.8%) were able to define this as the hepatitis B vaccine. There was an impressive number (50.2%) of HBV-unexposed individuals who had never taken the HBV vaccine. Among the 291 individuals without previous contact with HBV and who reported vaccination, only 27.5% were positive for anti-HBs. Prevalence of anti-HBs increased according to CD4 level, with a well-defined trend toward higher values at the higher CD4 levels (Table 4). Although precise information regarding the period of vaccination, the number of doses received, and the CD4 levels during the vaccination period was frequently difficult to obtain during interviews, the statistical association suggests that CD4 levels strongly influence vaccinal response to HBV. There are numerous reports describing impaired response to HBV vaccine in HIV-infected individuals [20,21]. A recently released double-blind, randomized study evaluated several doses of vaccine in 210 individuals and also found lower seroconversion rates among patients with CD4 <350 cells/mm^3 ^[22].

Low seroconversion rate in the HIV-infected population is a matter of concern, especially in patients with the lowest CD4 levels. Alternative strategies should be developed to improve anti-HBs production after vaccination, such as higher dosages of the vaccine, assessment of antibody levels after vaccination, increasing number of doses and revaccination after immune reconstitution. [22-24].

In conclusion, our data indicated that the HBV prevalence in HIV-positive population in the state of Mato Grosso is similar or slightly lower than that in other regions of Brazil. A less important role of drug injection in the spread of HIV and HBV in Mato Grosso was revealed when compared to other, more developed and populous regions of the country. However, it remains merely speculative if it affected co-infection prevalence.

Anyway, the low HBV vaccine coverage showed by the present survey should alert health care workers to the need of testing for HBV markers and reinforcing HBV vaccination when dealing with individuals living with HIV.

## Competing interests

The author(s) declare that they have no competing interests.

## Authors' contributions

All of the authors have contributed to the design of the protocol and the acquisition of data. ADHM and VCAS performed the immunoassays. All of the authors were involved in analyzing data. RARAP and FJDS drafted the initial manuscript. ADHM and VCAS revised it in order to improve the text.

## Pre-publication history

The pre-publication history for this paper can be accessed here:


